# The impact of COVID-19 on relationships between family/friend caregivers and care staff in continuing care facilities: a qualitative descriptive analysis

**DOI:** 10.1186/s12912-023-01289-7

**Published:** 2023-04-14

**Authors:** Emily Dymchuk, Bita Mirhashemi, Stephanie Chamberlain, Anna Beeber, Matthias Hoben

**Affiliations:** 1 Faculty of Nursing, College of Health Sciences, University of Alberta, Edmonton, AB Canada; 2 School of Nursing, University of North Carolina at Chapel Hill, Chapel Hill, NC USA; 3 School of Health Policy and Management, Faculty of Health, York University, Room 301E Stong College 4700 Keele Street, Toronto, ON M3J 1P3 Canada

**Keywords:** Long-Term care, Assisted Living Facilities, Nursing Homes, Health Personnel, Caregivers, COVID-19, Professional-Family Relations

## Abstract

**Background:**

The COVID-19 pandemic and related public health measures added a new dynamic to the relationship between caregivers and care staff in congregate care settings. While both caregivers and staff play an important role in resident quality of life and care, it is common for conflict to exist between them. These issues were amplified by pandemic restrictions, impacting not only caregivers and care staff, but also residents. While research has explored the relationship between caregivers and care staff in long-term care and assisted living homes, much of the research has focused on the caregiver perspective. Our objective was to explore the impact of COVID-19-related public health measures on caregiver-staff relationships from the perspective of staff in long-term care and assisted living homes.

**Methods:**

We conducted 9 focus groups and 2 semi-structured interviews via videoconference.

**Results:**

We identified four themes related to caregiver-staff relationships: (1) pressure from caregivers, (2) caregiver-staff conflict, (3) support from caregivers, and (4) staff supporting caregivers.

**Conclusions:**

The COVID-19 pandemic disrupted long-standing relationships between caregivers and care staff, negatively impacting care staff, caregivers, and residents. However, staff also reported encouraging examples of successful collaboration and support from caregivers. Learning from these promising practices will be critical to improving preparedness for future public health crises, as well as quality of resident care and life in general.

**Supplementary Information:**

The online version contains supplementary material available at 10.1186/s12912-023-01289-7.

## Background

The COVID-19 pandemic added an unprecedented dynamic to the relationship between family/friend caregivers and care staff in congregate care (i.e., long-term care and assisted living) homes [1–7]. Family/friend caregivers (who in the following we will consistently refer to as *caregivers*) are individuals (e.g., relatives, partners, friends, neighbours) who have a significant personal relationship with a congregate care resident and provide assistance to that person [8, 9]. Care staff working in congregate care settings include [10] (a) unregulated care workers (who go under many names, such as personal support workers or nursing assistants, and who we will consistently refer to as *care aides*), (b) regulated nurses (including licensed practical nurses and registered nurses), (c) allied health providers (e.g., recreational therapists, occupational therapists, speech language therapists, physiotherapists), and sometimes (d) others (such as clinical educators, quality improvement specialists, social workers). In addition, care unit managers and facility-level management (directors of care, facility administrators) are involved in interactions with residents and their families/friends. The quality of the relationship and communication between caregivers and care staff greatly influences resident, caregiver, and care staff health and wellbeing [11–14]. However, even before the COVID-19 pandemic, conflicts between caregivers and care staff were commonly reported [12, 14–17]. Caregivers often provide most of the care and support to the individual in need of care when they live in the community. When the care recipient is admitted to a congregate setting, caregivers want to remain involved in these care tasks [14, 15, 17]. However, their duties and responsibilities often shift. As care staff take on many of the physical care tasks, caregivers contribute more to resident care decisions, support resident involvement in meaningful activities, and maintain the resident’s connections outside the care home [16]. With a lack of formal policies regarding the role of caregivers, care staff and caregivers are left to navigate this transition on their own – leading to conflicts and dissatisfaction for both [12]. Most of the research on caregiver-staff relationships has focused on the perceptions of caregivers and only few studies have highlighted care staff perspectives on these issues.

Caregivers expect and appreciate open and consistent communication from staff on the resident’s condition and are reassured when staff listen and act on their concerns [11, 13, 15, 16]. Most care staff recognize the importance of regular interactions with caregivers, but they often feel that they lack time for these conversations [13, 16]. Staff may also believe that caregivers have unrealistic expectations given the realities they face when caring for multiple, high-need residents [13, 16]. Caregivers who are perceived as too demanding or questioning staff in their work generally have poorer relationships with care staff and care staff often further withdraw in their interactions with caregivers [13, 15]. This further increases caregivers’ mistrust who then feel the need to oversee care staff while they provide resident care – another common source of conflict between caregivers and care staff [11, 12, 14, 17]. Research suggests that these communication issues may prevent caregivers from passing on critical knowledge about the resident to care staff, possibly deteriorating resident quality of life [18].

The COVID-19 pandemic required health systems to implement various public health measures to protect frail, older adults living in congregate care homes, including isolation and visitor restrictions. While these measures protected residents from severe health risks, it limited caregivers’ ability to see and interact with residents. COVID-19 visitor restrictions diminished the critical contribution of caregivers to the quality of care and quality of life of residents [1, 2, 6, 7, 19]. While most homes offered alternatives to face-to-face communication, such as window visits or video calls, residents and their caregivers often perceived them as poor substitutes for in-person interactions, especially if the resident’s cognition was impaired [1, 2, 4, 7, 19–21]. Caregivers were forced to rely on management and staff to receive updates on the resident’s condition [2, 6, 22], substantially increasing caregivers’ own anxiety and depression [19, 23], as well as their concerns about the resident [5]. In turn, staff were tasked with organizing virtual visits and facilitating regular resident updates for caregivers – tasks that were added to the already elevated workload due to the COVID-19 pandemic (e.g., ensuring physical distancing of residents, putting on personal protective equipment, hygiene measures) and in a situation of severe staffing shortages due to one-site staffing policies [21].

While many caregivers acknowledged the challenges staff faced while navigating the pandemic [1–3], emerging evidence suggests that they also increased pressure on care staff because they felt communication was missing or insufficient [1, 3–7]. Caregivers worried about the decline in residents’ mental and physical health [3, 5–7, 20, 22], and experienced uncertainty of whether the resident received appropriate care [1, 4, 19]. Many caregivers also expressed frustration in how essential visitor, social distancing, and masking policies were applied by care homes [2, 6, 20, 21]. Inconsistencies among care homes led to confusion for many caregivers [2, 6, 20] as they struggled to understand why they had limited access to the residents while staff could be close to residents, even if staff’s vaccination status was unknown [21].

While the perspectives of caregivers are important when exploring the relationship between caregivers and care staff, it is also critical to recognize the perceptions of care staff to understand and improve these caregiver-staff relationships. Therefore, in this study we explored the perspectives of site administrators, directors of care, and front-line staff (care aides, nurses, allied health providers) from long-term care and assisted living homes in Alberta, Canada as part of a larger program of research evaluating the impact of COVID-19 on residents living in congregate care homes and on their paid and unpaid caregivers. Specifically, this paper seeks to explore the impact of COVID-19-related public health measures on caregiver-staff relationships from the aforementioned stakeholders’ perspective.

## Methods

### Ethics approval

This research was approved by the University of Alberta Research Ethics Board (Pro00096355) and we obtained operational approvals as needed from participating health authorities and congregate care settings. Informed verbal consent was obtained from each participant and documented prior to the start of the focus groups and interviews.

### Study design

The qualitative descriptive content analysis [24] presented in this paper is derived from focus group data collected in a larger study, comprehensively assessing the impact of COVID-19 on the health and wellbeing of residents, caregivers, care staff and managers.

### Study setting

We recruited a convenience sample of six long-term care and five assisted living homes in Alberta, Canada. In Alberta, long-term care homes are publicly subsidized and regulated institutional settings that provide 24-hour care to residents with complex care needs and have a registered nurse on site [25]. Alberta’s publicly subsidized and regulated assisted living settings are called Designated supportive living. Like long-term care, they are congregate care settings, however, they do not offer 24-hour on-site registered nursing care [26, 27]. Designated supportive living instead provides access to care from licensed practical nurses (with registered nurses on call) and these settings are divided into three levels (designated supportive living 3, 4, and 4D for individuals with dementia) depending on the degree of assessed care needs [28].

### Study sample

We recruited a convenience sample of a broad range of staff from each of the participating long-term care and assisted living homes. Participants included site administrators, managers, clinical educators, social workers, licensed practical nurses, registered nurses, care aides, recreation and rehabilitation staff, as well as housekeeping and dietary staff. Participants also varied in age, gender, level of education, and years of experience in their role. The study team worked with a key contact (usually a director of care or a facility administrator) to identify and recruit a variety of staff in each home to ensure a broad range of perspectives.

### Data collection

Between July 2020 and April 2021, we conducted a total of 9 focus groups [29] (one in each of 9 of the 11 participating homes). For the remaining two homes, only one of the invited participants dialed in to the scheduled data collection, so we conducted a semi-structured interview [30] instead of a focus group. All focus groups and interviews were conducted via Zoom^®^ due to COVID-19 restrictions. We scheduled focus groups based on site and participant availability. The number of participants per focus group ranged from two to eight (which was mostly within the general recommendations of focus group sizes between 4 and 12 participants [29]), and the meeting duration ranged from 41 to 59 min.

Focus group participants were recruited via the main contact at each site (usually an administrator or care manager). We established a relationship with this individual prior to conducting the focus group by introducing the study team and discussing the reason for the study, the study aims and processes. We encouraged the main contact to invite all members of their staff, including health care aides, nurses, recreation and rehabilitation staff, and educators, to participate in the focus group discussion. However, we emphasized that participation of management and care staff was voluntary and asked managers not to exert pressure on care staff to participate. We highlighted to managers that staff who attended focus groups involuntarily would likely not speak up at all or only share information they felt was safe to share – limiting the usefulness of insights gained in the focus groups for the researchers and the facility. We emphasized these facts again at the start of each focus group and completed a verbal informed consent with each participant. We specifically pointed out the option not to speak or to leave the focus group to participants. The staff who participated in each focus group differed by facility depending on staff availability and interest.

We created a focus group guide with questions related to how COVID-19 health measures impacted the home, challenges and strategies related to implementing these measures, and how they impacted residents, caregivers, and staff (see Additional File 1). Focus groups centred mainly on the first two waves of the COVID-19 pandemic as the first lockdowns began in Alberta in March 2020, with the second wave re-emerging in November 2020 into spring 2021.

Either the study’s Principal Investigator (MH, male, RN, Dr rer medic in Health and Nursing Sciences), or Research Coordinator (ED, female, BSc in Human Ecology) led the focus groups. Both are trained and have experience in leading focus groups. The facilitator and other study team members present during the focus group introduced themselves to the focus group participants, shared their roles on the study team and during the focus group, their professional backgrounds and their personal motivation to be part of the study team and help carry out the study. Informed consent was obtained verbally with each participant via Zoom^®^ before the start of each focus group. A member of the research team documented their consent electronically. Participants were encouraged to share their perspectives openly and were asked to keep anything discussed in the focus group confidential. We recorded the focus groups for transcription purposes. Recordings were transferred and stored in a secure repository at the University of Alberta. Participants were invited to complete a short demographic survey (Additional File 2) following the focus group.

### Data analyses

We transcribed focus group recordings verbatim using Word, and used thematic analysis [31]. We created a preliminary coding scheme before the analyses based on the focus group questions. For example, the focus group question “Were there any challenges you encountered implementing the measures?” was developed into the code “implementation challenges.” We then developed additional codes inductively based on the themes discussed by participants (i.e. the code “pressure from families//friends” was created as a subcode under “implementation challenges”). Three members of the study team independently analyzed and coded the transcripts using Quirkos [32]. Three team members (ED, BM, HK) independently coded batches of three transcripts at a time and then met virtually to discuss the codes, reconciling and refining the coding scheme. In addition, the whole team met regularly to discuss the codes, themes developed, and interpretations. During individual coding, as well as during the reconciliation and team meetings, team members created memos documenting their thoughts and interpretations, and comprehensively documented all decisions made throughout the study. This process of investigator triangulation is a well-established practice to ensure rigor and trustworthiness of qualitative research [33]. No new themes emerged after 5 of the 9 focus group transcripts were coded, suggesting that our sample size was sufficient to achieve data saturation. Due to the COVID-19 pressures experienced by care teams, we were not able to reach out to each team individually for member checking of our findings. However, we presented our study findings to care teams in two webinars, receiving input that helped us further contextualize and refine our codes and interpretations.

## Results

A total of 37 individuals from six long-term care and five assisted living homes participated in the data collections. Of those, 33 (89%) provided demographic data (Table 1). Most identified as women (88%). Ages ranged from younger than 30 years to over 60 years. Most participants had obtained a post-secondary education and 15 (41%) participants identified their role in the facility as “other”, which included recreation and rehabilitation staff, social workers, and quality practice leads. The mean (standard deviation) number of years worked in the current role was 6.4 (± 8.9).

**Table 1:** Demographic characteristics of participating care staff (n = 37)

Characteristic	N (%)
Gender, n (%)	
Male	4 (11%)
Female	29 (78%)
Missing	4 (11%)
Age Range, n (%)	
≤ 30 years	5 (14%)
31–40 years	7 (19%)
41–50 years	7 (19%)
51–60 years	4 (11%)
> 60 years	10 (27%)
Missing	4 (11%)
Education Level, n (%)	
High school diploma or diploma/certificate	12 (32%)
Bachelor’s degree	14 (38%)
Master’s degree	7 (19%)
Missing	4 (11%)
Setting	
Long-term care	27 (73%)
Assisted living	10 (27%)
Role in Facility, n (%)	
Facility administrator	3 (8%)
Director of care	4 (11%)
Care manager	7 (19%)
Clinical educator/specialist	4 (11%)
Care staff (licensed practical nurse, care aide)	3 (8%)
Other	15 (41%)
Missing	1 (3%)
	**M (SD)**
Years worked in current position	6.4 (8.9)

We identified four themes related to caregiver-staff relationships: [1] pressure from caregivers, [2] caregiver-staff conflict, [3] support from caregivers, and [4] staff supporting caregivers. In the following, we present each of these themes and related sub-themes. Supporting quotes from participants are in italic fonts. Participant IDs at the end of each quote reflect the participant’s role (CM: care manager, CES: clinical educator/specialist, CS: care staff, DOC: director of care, FA: facility administrator, O: other), the mode of data collection (FG: focus group, SSI: semi-structured interview), and the number of the participant (P1-P37).

### Theme 1: pressure from caregivers

The experiences shared by care teams within this theme reflected 4 sub-themes, including [1] managing contradictory demands from different groups of caregivers, [2] interacting with caregivers who question, bend, and break the rules, [3] keeping up with changing public health measures and related caregiver demands, and [4] managing caregivers’ worries and anxiety.

#### Managing contradictory demands from different groups of caregivers

Staff at both long-term care and assisted living homes reported mixed reactions from caregivers on the implementation of COVID-19-related public health measures. Management at one long-term care home spoke about their experience early in the first wave:

*“families who were demanding us close down or they were going to report us, even though it wasn’t required. And families begging us not to close.”* FA-FG1-P1.

This put management in a difficult position as they tried to balance the quality of life and safety of residents while managing the pressure from caregivers. This particular home ultimately made the decision to close their doors to visitors prior to any ministerial orders due to the *“pressure from a small vocal minority”* (FA-FG1-P1). Regardless of this decision, management felt that they had been *“fairly good at providing access to the residents’ families”* (FA-FG1-P1) throughout the first wave of the pandemic. They also acknowledged the impact on resident quality of life as some residents were noticeably more restless after the home closed its doors for the first time. A member of the home’s leadership team shared her observation of some of the residents’ *“aloneness”* (sic.) (O-FG1-P3), especially those with caregivers who were previously there on a regular basis. On the other hand, staff also observed improvements in some residents, particularly those with cognitive impairment, as a result of fewer visitors being present:

*“Over a little bit of time, we actually kind of thought some residents did better. Less activity. Less hubbub. Less coming and going. We saw some settling.”* CM*-*FG1-P2.

#### Interacting with caregivers who question, bend, and break the rules

Staff at one assisted living home encountered caregivers who insisted on seeing the resident in the home, regardless of the essential visitor designation throughout the first year of the pandemic. The director of care at the home recalled a caregiver who questioned the site’s essential visitor designation approval process and repeatedly came to see a resident. Although the staff had not authorized this individual to visit, and had perceived this particular resident as managing well with the COVID-19 measures in place, they allowed the individual to visit their loved one for a *“one hour maximum”* (DOC-SSI2-P19). The caregiver would then *“stay three hours, four hours”* (DOC-SSI2-P19), multiple days a week. This ultimately created additional pressures for staff who tried their best to ensure that measures were enacted to keep the residents safe.

#### Keeping up with changing public health measures and related caregiver demands

As COVID-19 public health measures evolved over time, caregivers were quick to call the care homes about changes to visitor restrictions. Care staff across multiple homes spoke about the fact that they learned of the government’s changes to the COVID-19 health measures at the same time as the public, and oftentimes had not even had a chance to review the order before being *“bombarded with people”* (O-FG9-P36) calling about visiting residents. This was especially difficult for staff when the care homes were given the autonomy to implement greater restrictions if they felt it was necessary but caregivers disagreed with these measures. Staff at one long-term care facility noted the pressure they felt in this situation as COVID-19 cases reached record highs in the second wave:

*“It’s nerve racking really because we want our residents to… we want them to be able to see their loved ones, and we want their loved ones to be able to see them. At the same time, you know the cases in the community are higher than they’ve ever been.”* O-FG9-P36.

#### Managing caregivers’ worries and anxiety

At times, caregivers’ worries increased their expectations for care staff. A care aide at one assisted living home shared that some caregivers questioned their actions and became *“more demanding than they would normally be”* (CS-FG4-P14) towards staff as they entered the second wave. However, this individual and their manager also recognized that this was not personal:

*“Some people just – it is their personality to be anxious and high strung. So if they bring it to us, we know that it isn’t personal. It isn’t that, you know, the person that’s having to take it on, it’s not that they don’t like you or you’ve done something wrong necessarily. It’s just that people are coming to us with their own stressors out of the community and you might be maybe the only other person they’ve seen in, you know, a number of days or weeks.”* CM-FG4-P13.

Staff at a long-term are home had a similar outlook when dealing with unsatisfied caregivers during the first and second wave:

*“there were definitely frustrations, but at the end of the day you just have to you know, put your best foot forward, don’t take it personally right…it’s not against you personally, it’s everything as a whole.”* DOC-FG8-P28.

This perspective likely helped to maintain relatively positive caregiver-staff relationships.

### Theme 2: staff-caregiver conflict

Experiences reported by care teams within this theme reflected 3 sub-themes, including [1] conflicts with caregivers who disagree with or bend/break the rules, [2] caregivers’ verbal aggression toward staff, and [3] caregiver accusations toward staff.

#### Conflicts with caregivers who disagree with or bend/break the rules

The ways in which public health measures were implemented across care homes contributed to tensions between caregivers and staff. Throughout the pandemic, some caregivers refused to follow the rules of the care homes, avoiding screening or hiding potential COVID-19 symptoms they were experiencing in order to visit the resident. As management at one long-term care home said:

*“Some family members are coming in sick or they’re not being upfront and truthful with us…they thought we were overreacting.”* DOC-FG2-P6.

This resulted in staff having to confront these individuals, even utilizing a security guard at the entrance of the home to protect the staff and residents during the first wave. Staff at another long-term care home echoed similar experiences throughout the first and second waves and felt like they were judged and labelled as *“bad guys and not following the orders”* (CM-FG9-P35) by caregivers if they chose to implement stricter guidelines than outlined by the government. A social worker at the home spoke further about this issue and the impact on resident quality of life:

*“I wish that the public health would just take over and just dictate the rules because it’s hard for us as an organization to be able to, you know, justify our restrictions and stuff to families, because we’re sort of always put in this really awkward position of, you know, we’re erring on the side of like extreme caution and so we’re always sort of balancing that safety and quality of life.”* O-FG9-P36.

Looking back to the first year of the pandemic, the director of care at one assisted living home spoke of caregivers *“challenging”* (DOC-SSI2-P19) and fighting with the staff, accusing them of unreasonable rules and restrictions. This coincided with some caregivers bending the rules, such as bringing in homemade food for residents, or gathering in the home’s parking lot when the weather allowed during the first wave. This caused confusion and frustration for staff as well as negatively impacted the quality of life of other residents in the home. As the director of care shared, *“it increased the anxiety level”* (DOC-SSI2-P19) of residents when they witnessed others breaking the rules. Residents appeared to be *“deeply concerned”* (DOC-SSI2-P19) being put at risk for contracting COVID-19.

#### Caregivers’ verbal aggression toward staff

At times, care staff felt caregivers reacted inappropriately towards them, yelling at them and disregarding their roles. One care aide expressed:

*“people can definitely use the staff as a whipping post.”* CS-FG4-P14.

This added to the feelings of stress the care aide was already experiencing as they entered the second wave of the pandemic. A number of caregivers communicated their frustration of not being able to visit the resident in person, while staff were allowed to be close to them. One participant recalled the difficulty in rationalizing the restrictions to caregivers during the first wave:

*“It was really hard to explain to the families who say “I’m not allowed to touch my mother, but you can? And you go out into the public. Why is that?” I couldn’t answer that question. It’s all I could say was “That’s the order. I’m sorry.” It didn’t make any sense to me either, why, how come I can touch them.”* CM-FG9-P35.

#### Caregiver accusations toward staff

In some cases, caregivers accused care staff of bringing COVID-19 into the home and infecting residents. This was upsetting to one social worker who knew many of the staff at the assisted living home had actually limited their activities in their personal lives during the first wave and as they moved into the second wave of the pandemic:

*“We’ve heard from care staff who restrain themselves, basically, to keep the residents safe and didn’t go to restaurants or anything at all anymore.”* O-FG5-P16.

This was echoed across several homes, and some staff even lived away from their own families in order to reduce their risk of contracting COVID-19. Staff at one long-term care home felt an overall lack of acknowledgement from caregivers on the work they were doing to protect the residents and care environment throughout the pandemic. A staff member shared:

*“There’s not been a lot of appreciation for that, of what it takes to actually do this and do it right and do it safely.”* O-FG9-P36.

These examples reflect opposing feelings between caregivers and care staff regarding the ways in which the COVID-19 public health measures were implemented at these homes.

### Theme 3: support from caregivers

Caregiver experiences under this theme encompass 3 sub-themes, including [1] caregivers’ display of gratitude and appreciation, [2] caregivers making material or financial donations, and [3] creating caregiver commitment by shared decision making.

#### Caregivers’ display of gratitude and appreciation

Although care staff perceived some caregivers to be dissatisfied and lacking appreciation for staff, there were various reports of situations in which care staff experienced caregivers as being grateful for their work. As one assisted living staff member said:

*“They were just rooting for us and it was so heartwarming.”* CES-SSI1-P12.

This reaction from caregivers was largely in response to the staff’s work during the home’s COVID-19 outbreak during the first wave. In order to support the efforts of care staff, some caregivers chose to not visit the home, even when restrictions allowed. A care manager at one home shared:

*“We’ve asked people to stay away for so long and many of them have – some people voluntarily because they recognized the risk. Maybe of their own lifestyles in the community because there’s many, you know, people here who have children that are still in the workforce… most of the families here, I think, have been very understanding of these measures and, you know, understanding that they need to really be mindful of their own risks within the community.”* CM-FG4-P13.

Such displays of understanding and appreciation from caregivers early in the pandemic were very encouraging for care staff; however, the care manager also acknowledged *“the importance of the family connections”* (CM-FG4-P13) and further recognized *“that people’s quality of lives will continue to deteriorate without those connections”* (CM-FG4-P13), including that of residents.

#### Caregivers making material or financial donations

It was also common for caregivers to donate to the care homes, whether it be electronics, such as tablets, or financially to some of the homes’ foundations. Such donations were extremely helpful to ensuring staff could assist residents to engage with their caregivers outside of the home all through the pandemic.

#### Creating caregiver commitment by shared decision making

Staff at one assisted living home spoke about involving residents and caregivers in decision making related to COVID-19 from the very beginning of the pandemic. This led to residents and their caregivers being *“supportive with the decision of not taking residents out or not coming [in]”* (DOC-FG3-P8). Such involvement also led to care staff and caregivers to get to know each other better. One nurse shared:

*“It gave us an opportunity to kind of like communicate more with the families, right? Opposed to like most of the time, they’ll just come into the facility, visit, and then they’re out. But then you get to know more people – more of the families on hand because they’re communicating more with us and like ‘how is my mom? Or how is my dad doing?’ Right?”* CS-FG3-P10.

Such interactions seemed to improve the caregiver-staff relationship.

### Theme 4: staff supporting caregivers

This theme comprised care team experiences in 3 sub-themes, including [1] ongoing, open, and encouraging communication with caregivers, [2] Providing information, education, and resources to caregivers, and [3] making structural changes to accommodate caregivers.

#### Ongoing, open, and encouraging communication with caregivers

All of the care homes reported implementing strategies early in the pandemic to provide support to residents’ caregivers. One of the key strategies was ongoing, open communication. Most care staff spoke of weekly messages via email, newsletter, or social media, to caregivers to provide updates on the home’s approach to the COVID-19 pandemic. Management at one long-term care home reflected:

“*We try to do – have a consistent approach of what’s happening in the building as far as COVID. What are some updates in the building? And then always some good news, you know, some of the great things that are happening. And then just some encouragement and inspiration at the end of the message because regardless of where we find ourselves during this COVID period, it’s impacting everybody. So just [Unclear] to try and, you know, keep everybody’s spirits positive.”* FA-FG1-P1.

Staff at another long-term care home also mentioned regular phone calls as a *“wonderful opportunity to bond with families”* (O-FG8-P34). In order to do this, staff oftentimes worked longer hours or came in on weekends throughout the first and second waves of the pandemic. Many staff also shared their personal contact information with residents’ caregivers to increase their ability to reach them.

#### Providing information, education, and resources to caregivers

Staff across most of the care homes spoke about providing education and resources to residents’ caregivers. Management at one long-term care home talked about letting caregivers know *“what COVID was”* (DOC-FG2-P6) early on, as well as *“teaching them how to don and how to doff”* (DOC-FG2-P6) when referring to gowns, masks, and gloves, while visiting the resident. Most of this took place before government issued mandates were even in place. Staff at another home shared their experiences of offering caregivers resources related to self-care during the first and second waves of the pandemic:

*“You know, five days a week, they were doing Facetime and then they wanted to visit in times like you need something else in your life, other than mom and COVID, so it was kind of neat to see how we could help them find some healthier… I guess caregiver care, you know?”* O-FG8-P34.

Further, a social worker at a long-term care home talked about providing *“supportive counseling to family members who were struggling with what was happening”* (O-FG9-P36) in regards to a COVID-19 outbreak at the home during the first wave. Caregivers were also taught how to use technology, such as Facetime^®^ and Zoom^®^, in order to virtually connect with the residents.

#### Making structural changes to accommodate caregivers

Some homes even rearranged rooms to assist caregivers in visiting the resident via window visits, or inside when permitted. For example, one long-term care home repurposed their hair salon into a space for caregivers to say goodbye to those residents who were at end of life:

*“[We] made this hair salon into a room. And we chose that room because it had a window. We didn’t want people passing away to be without their loved ones. We kind of made it like an ICU room. Sometimes people may not be comfortable going in the room, but at least they could see their loved ones.”* DOC-FG2-P6.

These examples illustrate the importance staff placed on their relationship with residents’ caregivers as they sought to support them during the COVID-19 pandemic. As one long-term care home administrator said:

*“It’s been the investment in our families, the investment in our staff, and investment in each other is what really got us through.”* FA-FG7-P25.

## Discussion

This study addressed an important knowledge gap – perceptions of managers and staff in long-term care and assisted living homes on the impact of the COVID-19 pandemic on their relationships with caregivers. While care teams reported on the challenging aspects of their relationship with caregivers, they also shared moments of cooperation and unity, reflecting a balanced point of view. Specifically, care teams reported many challenges they faced as caregivers’ demands and behaviours put pressure on care teams, and often led to various conflicts between care teams and caregivers. However, care teams also provided multiple examples of relationships gone well, when caregivers supported care teams and care teams provided support to caregivers.

### Comparison of study findings with other studies assessing the impact of COVID-19 on caregiver-staff relationships

Studies assessing the impact of the COVID-19 pandemic on managers [34, 35], nurses [36, 37], and care aides [36, 38] in long-term care more broadly support our findings. While these studies did not specifically focus on the impact of COVID-19 on the relationships between care teams and caregivers, some report related results. Like our study, Savage et al. [35] found that according to 21 managers in eight long-term care homes in Western Canada, implementing rapidly changing public health measures that often lacked detail, compromised longstanding and trusting relationships with caregivers (reflecting our themes 1 and 2). For example, when health authorities announced the implementation of outdoor visits, caregivers expected immediate implementation, while care teams required more time to plan and organize the details. Managers and staff were required to perform both regular and pandemic-related duties – often under conditions of severe staffing shortages [34, 35, 37, 38]. Kyler-Yano et al. [34] conducted semi-structured interviews with 40 assisted living administrators in Oregon, USA. They found that these individuals not only had to manage their own and their staffs’ emotions, but also the residents’ and caregivers’ emotions, leading to significant impacts on the administrators’ own mental health and well-being (reflecting our theme 1, especially the sub-theme managing caregivers’ worries and anxiety). A Canadian qualitative interview study including 52 care aides from nine long-term care homes [38] found that having to explain public health measures (e.g., the requirement to wear a mask) to caregivers multiple times a day – often meeting caregivers’ reluctance and resistance – profoundly wore on care aides (reflecting our sub-themes of interacting with caregivers who question, bend, and break the rules). Ahokas and Hemberg [39] describe, based on qualitative interviews with eight managers of older adult care settings, how these issues caused substantial moral distress among the managers. Caregivers acting in ways that the managers thought were harmful to residents and staff (and often against what the managers thought were the residents’ preferences) led to moral conflicts, and managers often felt they were caught in the middle, unable to reconcile these differences. Comparable problems have been reported before the COVID-19 pandemic [40, 41], but the pandemic has amplified these issues in unprecedented ways.

Despite all the difficulties they faced, care teams participating in our study also reported various positive experiences of how care staff, residents and caregivers worked well together to overcome the challenges. This is a finding much less commonly reported. We only found one other study [42] that also reported positive experiences related to staff-caregiver relationships, found in a quote of one advanced practice clinician who mentions good cooperation among staff, residents and caregivers. In their qualitative study, involving care staff of one Canadian long-term care home, Hung et al. [36] found that the absence of caregivers strengthened relationships between residents and care staff who worked to compensate for the absence of caregivers during the visitor restrictions.

### Implications for nursing practice, research and policy

Promising practices and stories of success in managing the challenges imposed by the COVID-19 pandemic are an important knowledge gap. These stories exist, but systematically identifying them and making them visible will be key to improving the response to future public health crises. Our study demonstrates how care teams struggled with multiple pressures exerted by caregivers, often leading to conflicts, and often being based in caregivers’ concerns and anxiety. Our study also provides multiple examples of how care teams successfully navigated these challenges. Specifically, including caregivers proactively and consistently in the development of policies and activities that influence the care of residents is critical in developing trust and two-way communication. Involving caregivers in decision making on COVID-19 restrictions resulted in understanding and support for these rules, and prevented the implementation of policies that were based on the opinions of a vocal minority of caregivers. Two other studies by our team demonstrated that caregivers who felt well-informed and involved in resident care by care teams were less likely to be concerned about the resident’s health and wellbeing [43], and had lower risk of mental health issues (symptoms of depression and anxiety [23]). Nurses and care aides are the care providers closest to residents and caregivers, and many of the activities that helped care teams successfully navigate challenges are directly within the scope of practice and expertise of these care providers – including informing, educating and supporting caregivers [44], and advocating for residents [45]. However, nurses often struggle with including these activities in their daily care practices at the best of times [44, 45], and the crisis that unfolded during the COVID-19 pandemic created adverse context conditions and affected nurses’ mental health severely [34–38], further limiting their ability to take on these essential care activities. This clearly highlights the need to provide nurses and care settings with sufficient resources and mental health supports to enable the provision of client- and family-centred care.

### Limitations

This study is one of the few to assess care staff perspectives of how the COVID-19 pandemic and related visitor restrictions have impacted the relationships between care staff and caregivers. We included participants in a variety of roles, with various backgrounds and levels of experience, and working in long-term care homes as well as assisted living homes. However, we only included participants in Alberta, Canada. It is possible that these views may differ from staff in other Canadian provinces or territories or in other countries. Focus groups were held during the first and second waves of the COVID-19 pandemic. When analyzing the data, it was sometimes difficult to tell what period of time participants were referring to. It is also possible that staff perspectives could have changed following these time points as the COVID-19 public health measures evolved.

Due to the COVID-19 restrictions, the study team was not able to enter the participating care homes or speak with staff in-person. We often relied on a key contact at each home, usually an administrator or care manager, to reach out to staff to participate in the focus groups. Although we encouraged all levels of staff to be included, it is possible that some may have been left out for reasons beyond our control. Despite this, many of the focus groups did include a mix of staff from various levels within the care home. Involving front-line staff and managers in the same discussion may have caused some staff to withhold information or refrain from sharing their opinions in the presence of management, however, some of the strongest statements came from staff who participated in a focus group with their supervisors.

It is important to reiterate that we have only included staff’s perceptions of their relationships with caregivers during the pandemic. This is an important step in understanding this complex relationship, however, a contrasting exploration of care staff, caregiver and resident perspectives on the same situation will be critical in future research.

## Conclusions

Caregivers play a significant role in the lives of residents in congregate care, and their involvement, as well as their relationships with care staff significantly influence resident quality of life and care. The COVID-19 pandemic disrupted long-standing relationships between caregivers and care staff, negatively impacting care staff, caregivers, and residents. However, staff also reported encouraging examples of successful collaboration and support. Learning from these promising practices will be critical to improving preparedness for future public health crises, as well as quality of resident care and life in general.

## Electronic supplementary material

Below is the link to the electronic supplementary material.

Supplementary Material 1

Supplementary Material 2

## Data Availability

The transcripts generated and analysed during the current study are not publicly available to ensure participant confidentiality. Access can be granted on request if the individual requesting access meets all the confidentiality requirements associated with HRDR access. Please contact the corresponding author for requests to access and review the study data and materials.
