# Supplementary material for: The impact of COVID-19 on relationships between family/friend caregivers and care staff in continuing care facilities: a qualitative descriptive analysis
Source: BMC Nurs. 2023 Apr 14;22:121. doi: 10.1186/s12912-023-01289-7 (PMC10102683; doi:10.1186/s12912-023-01289-7)
Supplement: Supplementary file 1 — Supplementary Material 1 [file 12912_2023_1289_MOESM1_ESM.pdf]

Focus group guide

Preamble

Thank you for participating in this focus group. We are talking to you because you are a facility administrator, director of care, care manager, clinical educator, or quality improvement specialist. In our study we are evaluating COVID-19-related public health measures that were mandated by health authorities, how these measures impacted the facilities and how facilities implemented these measures. Since participants may discuss private details in this focus group, we ask you that you keep anything discussed in the focus group confidential.

Interview Question Guide

Instructions to the interviewer

- There are main *numbered* questions. Try to proceed through them in the order they appear, but also give people the freedom to move beyond the boundaries of each question to some extent.
- The bullets under each main question are ***probes*** you can use if participants have not talked about these areas in their response to the main question.
- You can also ask additional ***probing*** questions as a follow to things you hear if you think this will provide additional insights regarding barriers and facilitators of DEMQOL-CH assessment processes and/or the organization of those processes.
- The key to good focus group facilitation is picking up on and probing when relevant comments are made (saying things like “tell me more about that”) and steering people back on track when they get off topic.

Questions and probes:

i. Briefly describe from your perspective how COVID-19-related public health measures evolved over time

- When did it all start?
- What measures came first, how did they change?
- How was your facility affected by COVID-19? Did you have an outbreak? How many staff members and/or residents were affected?

ii. How did your facility respond to COVID-19 and to the mandated public health measures?

- Were there any challenges you encountered implementing the measures?
- What support did you get implementing the measures?
- What strategies did you apply to mitigate challenges?

iii. How did COVID-19 and related measures impact your facility?

- How were residents affected?
- How were families affected?
- How were care staff affected?
- How were managers affected?

Thank you very much for participating in this focus group
