# Supplementary material for: The impact of COVID-19 on relationships between family/friend caregivers and care staff in continuing care facilities: a qualitative descriptive analysis
Source: BMC Nurs. 2023 Apr 14;22:121. doi: 10.1186/s12912-023-01289-7 (PMC10102683; doi:10.1186/s12912-023-01289-7)
Supplement: Supplementary file 2 — Supplementary Material 2 [file 12912_2023_1289_MOESM2_ESM.pdf]

Demographic survey

1. Please indicate your **highest** educational level (only select one option):

☐1 High school degree
☐2 Diploma/Certificate
☐3 Bachelors Degree
☐4 Masters Degree
☐5 PhD/PharmD

2. What is your **primary** role in the facility (select only one)?

☐1 Health Care Aide, Personal Care Attendant, Special Care Aide, Nursing Attendant
☐2 Licensed Practical Nurse
☐2 Clinical educator/specialist
☐3 RAI coordinator
☐4 Care manager
☐5 Director of care
☐5 Facility administrator
☐5 Medical director
☐5 Other (please specify) ____

3. Please indicate your age range according to the following groups:

☐1 ≤ 30 years
☐2 31-40years
☐3 41-50 years
☐4 51-60years
☐5 > 60 years

4. What best describes your gender?

☐1 Woman
☐2 Man
☐3 Prefer to self-describe: __________
☐4 Prefer not to answer

5. How long have you worked in your current position?

☐ Years ☐ Months
